# Supplementary material for: Higher‐order assemblies of oligomeric cargo receptor complexes form the membrane scaffold of the Cvt vesicle
Source: EMBO Rep. 2016 Jun 6;17(7):1044–60. doi: 10.15252/embr.201541960 (PMC4931565; doi:10.15252/embr.201541960)
Supplement: Supplementary file 1 — Expanded View Figures PDF [file EMBR-17-1044-s001.pdf]

Expanded View Figures

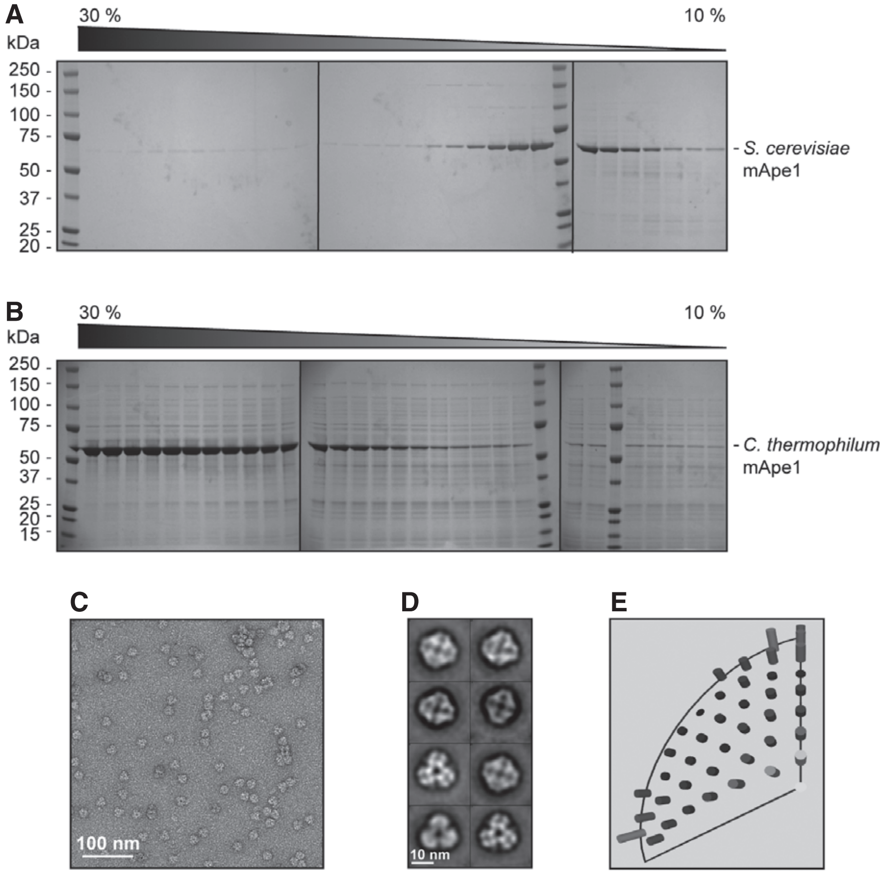

Figure EV1. Purification, negative stain analysis of *Chaetomium thermophilum* mApe1 (ct-mApe1) dodecamers, and comparison of ct-mApe1 with *Saccharomyces cerevisiae* mApe1 structure (related to Figure 1).

A, B Glycerol gradient (10–30%) of purified *S. cerevisiae* mApe1 (A), in comparison with (B) ct-mApe1, shows a shift toward larger oligomeric species of the thermophile protein indicating more stable oligomers. Samples were run on three separate gels.

C Negative stain electron micrograph of ct-mApe1 dodecameric particles.

D Class averages of ct-mApe1 dodecamers resembling those observed for mApe1 from *S. cerevisiae* in Fig 1E.

E Euler angle distribution of *S. cerevisiae* mApe1 EM structure.

 © 2016 The Authors

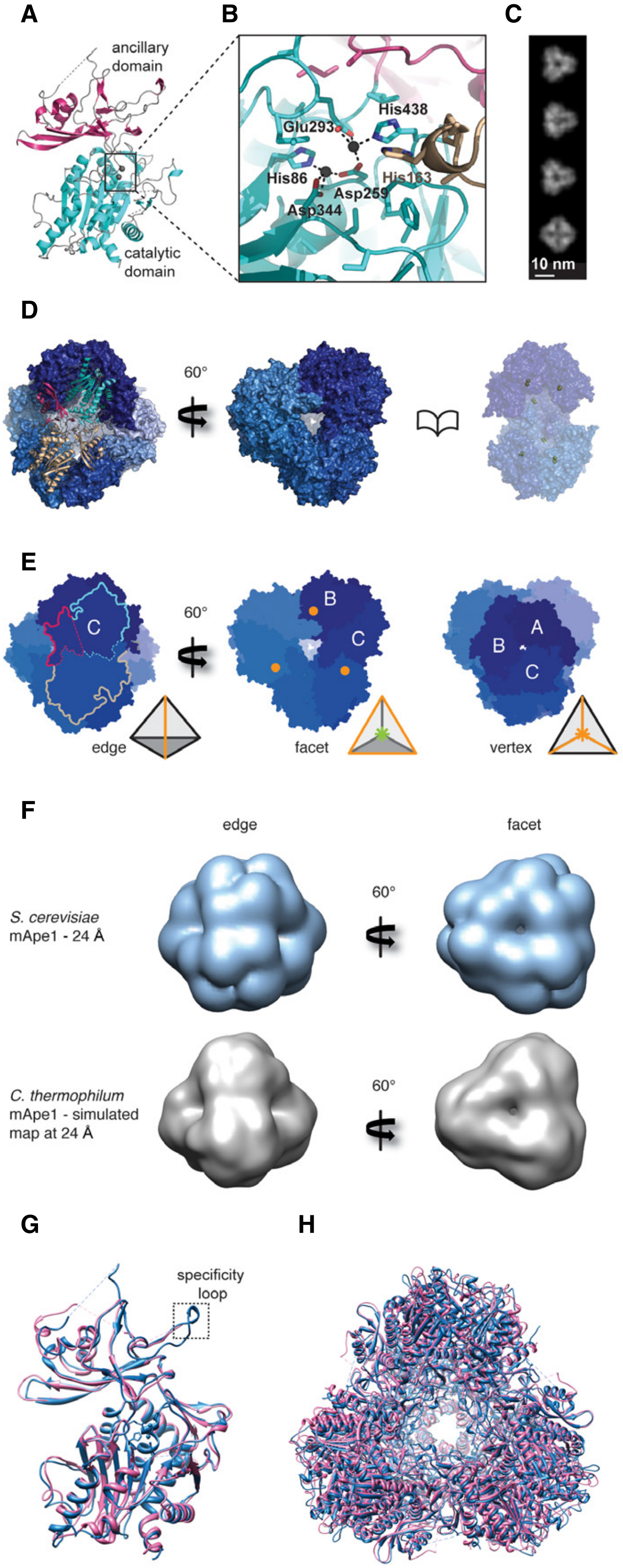

Figure EV2. *Chaetomium thermophilum* mApe1 crystal structure (related to Fig 1).

A Crystal structure of *C. thermophilum* mApe1 (ct-mApe1) at 2.8 Å resolution. Cartoon representation of ct-mApe1 structure including the catalytic domain (cyan) and the ancillary domain (magenta).

B Close-up view of the catalytic site. The binuclear Zn^{2+} center bridged by the bidentate ligand Asp259 of the active site is located at the top of the catalytic domain. A conserved histidine residue (His163) belonging to the neighboring subunit protrudes into the active site. Colors match with panels (A), (D), and (E).

C Reprojections of ct-Ape1 dodecamer X-ray structure with characteristic threefold and twofold axis views.

D Surface representation of the ct-mApe1 dodecamer viewed along the twofold axis on one of the tetrahedron edges. Two monomers (magenta/cyan and sand) forming dimers via interactions of their ancillary and catalytic domains are shown in ribbon representation (left). View along one of the facets (center). A book page view along one of the body diagonals reveals the catalytic sites (Zn^{2+}, yellow spheres) that are buried inside the hollow tetrahedral core (right).

E Tetrahedral symmetry of the ct-mApe1 dodecamer. Dimerization via the ancillary domains forms the edges of the tetrahedron (left). The characteristic views along the facet (threefold symmetry between dimers, center) and vertex (threefold symmetry between monomers, right) are also shown. The amino termini (orange circles) are located on the facets of the tetrahedron. Relevant monomers are labeled by letters for clarity.

F Comparison of twofold and threefold views (left and right) of the determined 24 Å resolution EM map of *Saccharomyces cerevisiae* mApe1 (sc-mApe1) (upper row) with the simulated 24 Å resolution map of the ct-mApe1 X-ray crystal structure (lower row) shows very close visual agreement.

G Superposition of ct-mApe1 monomer (blue) with sc-mApe1 monomer (pink) (PDB 4r8f) [31]. The Cα atoms have an RMSD of 0.7 Å.

H Superposition of ct-mApe1 dodecamer (blue) with *S. cerevisiae* mApe1 dodecamer (pink) (PDB 4r8f) [31]

© 2016 The Authors

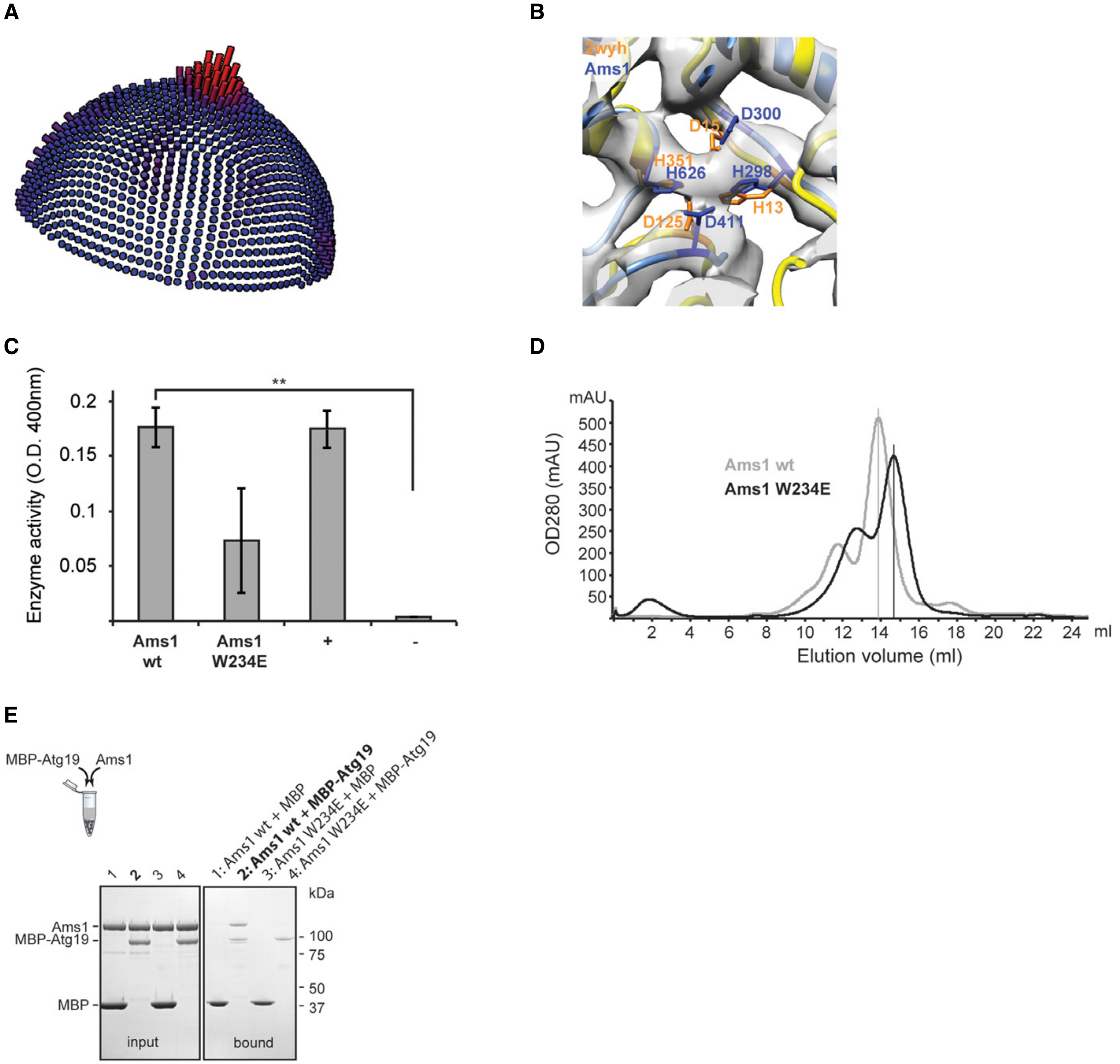

Figure EV3. Ams1 biochemical characterization of wild-type and W234E mutant (related to Fig 2).

A Orientation distribution of 6.3 Å Ams1 cryo-EM reconstruction.

B Putative catalytic site of Ams1 based on superposition of PDB 2wyh (orange) and Ams1 model (blue) fitted into cryo-EM density. The EM density is compatible with the catalytic residue arrangement of the *S. pyogenes* α-mannosidase. In analogy to the mApe1 structure, a putative Zn^{2+} ion is coordinated by central enzymatic residues of Ams1 His298, Asp300, Asp411, His626, and is located in the interior of the oligomeric assembly.

C Ams1 wild-type (wt) and W234E mutant preparations are catalytically active (positive control: Jack bean α-mannosidase; negative control: bovine serum albumin). Data are representative of three independent experiments and mean values are shown. Error bars indicate standard deviation (SD). $^{**}P = 0.003$ (Welch *t*-test).

D Size-exclusion chromatograms of wild-type Ams1 (gray) and W234E mutant (black) show a shift of the main peak toward smaller particles for the W234E mutant.

E SDS–PAGE of *in vitro* pull-down binding assay. Right: Purified wild-type Ams1 with MBP-Atg19 is captured on amylose beads (lane 2) whereas the W234E mutant does not show binding with MBP-Atg19 (lane 4). As controls, MBP does not bind wild-type Ams1 and W234E mutant (lanes 1 and 3). Left: SDS–PAGE showing the input of the corresponding pull-down assay.

 © 2016 The Authors

Figure EV4. Atg19 solubilization effect on prApe1 dodecamers (related to Fig 3).

A Size-exclusion chromatography profile (Superose 6 column) of Atg19 after anti-MBP-Atg19 purification and cleavage of the MBP tag (left). Shown in gray is the overlay with the profile of the prApe1/Atg19 complex after anti-MBP-Atg19 purification and cleavage of the MBP tag. SDS–PAGE of peak fractions of the gel filtration profile of Atg19 (right).
B Size-exclusion chromatography profile (Superose 6 column) of prApe1 after anti-GST-prApe1 purification and cleavage of the GST tag (left). Shown in gray is the overlay with the profile of the prApe1/Atg19 complex after anti-MBP-Atg19 purification and cleavage of the MBP tag. SDS–PAGE of peak fractions of the gel filtration profile of prApe1 (right). Samples were run on three separate gels.
C Representative SDS–PAGE of pelletation assay of purified prApe1 dodecamers alone, purified Atg19 alone, and the purified prApe1/Atg19 complex.
D Bar plot of gel band intensities from supernatant and pellet fraction of (C).
E Western blot of prApe1 (top) and Atg19 (bottom) showing clarified lysate from prApe1-GFP/Atg19-mCherry/ypt7Δ (lane 1) and prApe1-GFP/Atg19Δ/ypt7Δ (lane 2) *Saccharomyces cerevisiae* cells.

Data information: Data in (C) and (D) are representative of three independent experiments. Error bars indicate SD. Analysis of variance (ANOVA) was performed to assess whether the supernatant to pellet ratio of prApe1 changed upon the presence of Atg19 (D): ***$P = 1.7 \times 10^{-4}$.

© 2016 The Authors

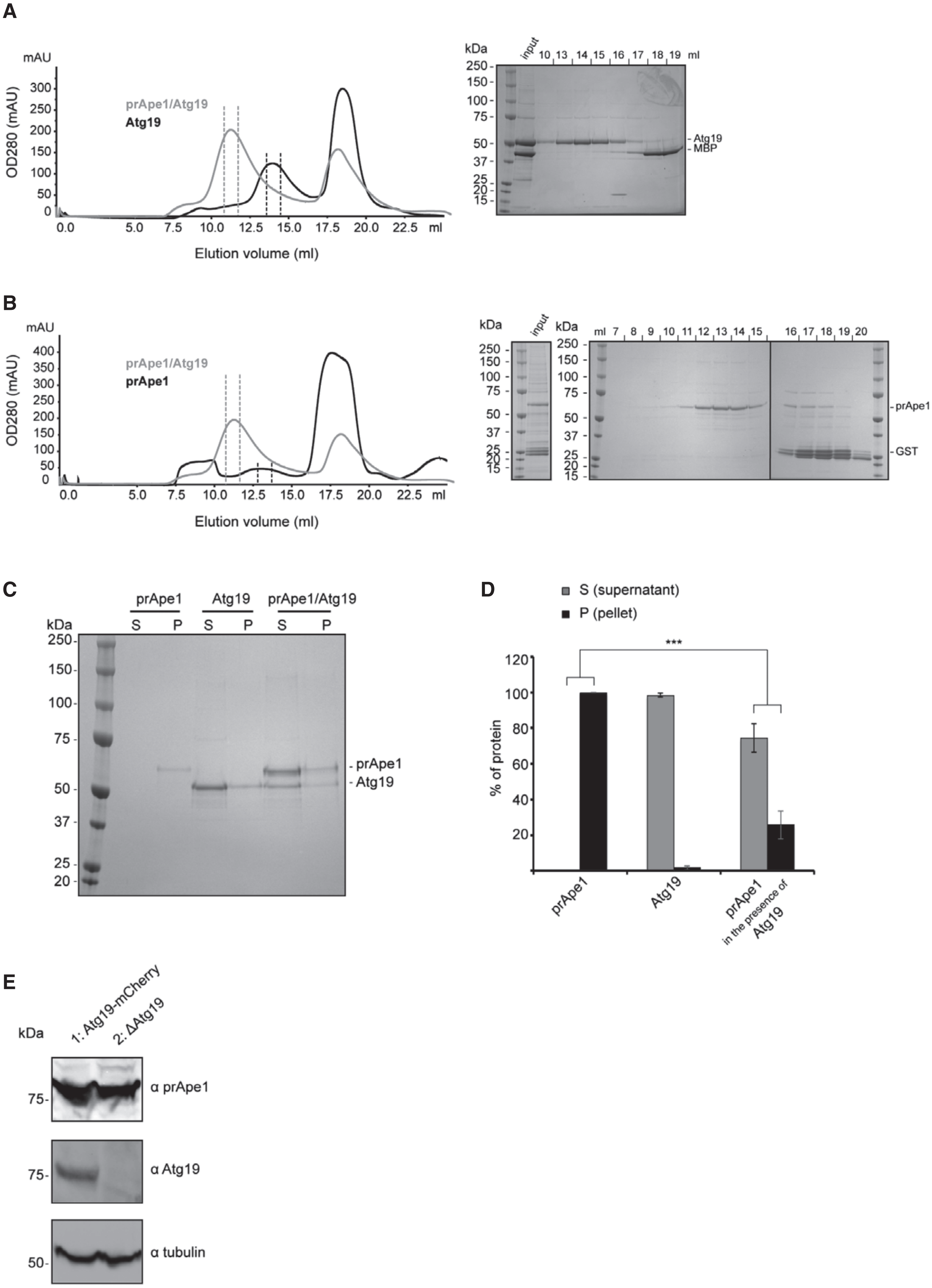

Figure EV4.

 © 2016 The Authors

Figure EV5. Tomographic slices of localized Cvt vesicles (related to Fig 5).

A–G prApe1-GFP/ypt7Δ *Saccharomyces cerevisiae* cells were high-pressure-frozen, resin-embedded, and sectioned. Gallery of tomographic slices showing the structures that localized to Ape1-GFP/ypt7Δ fluorescent spots.

H–O prApe1-GFP/Atg19-mCherry/ypt7Δ *S. cerevisiae* cells were high-pressure-frozen, resin-embedded, and sectioned. Tomographic slices showing the structures that colocalized to Ape1-GFP/Atg19-mCherry/ypt7Δ fluorescent spots.

Data information: White arrowheads show the double membrane. Circles in (G) and (O) show ribosome-like density. Scale bars, 100 nm.

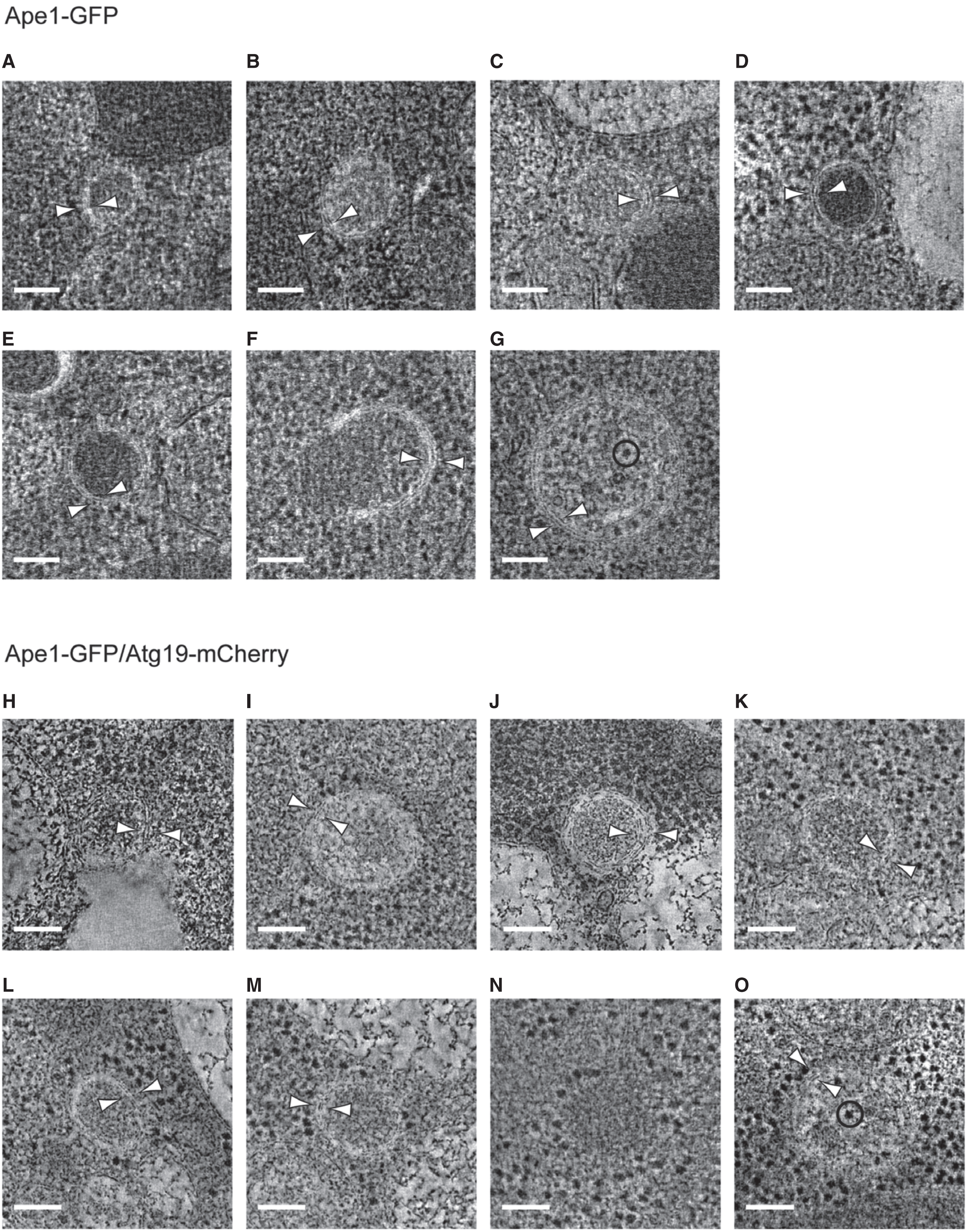

Figure EV5.

© 2016 The Authors
