# Supplementary material for: Higher‐order assemblies of oligomeric cargo receptor complexes form the membrane scaffold of the Cvt vesicle
Source: EMBO Rep. 2016 Jun 6;17(7):1044–60. doi: 10.15252/embr.201541960 (PMC4931565; doi:10.15252/embr.201541960)
Supplement: Supplementary file 2 — Table EV1 [file EMBR-17-1044-s002.docx]

Table EV1 List of yeast strains generated

| Strain | ID | Genotype |
| --- | --- | --- |
| Ape1-GFP/ypt7Δ | Ape1-GFP | MATa, *his3-Δ200*, *leu2-3,112*, *ura3-52*, *lys2-801*, Ape1*-GFP::HIS3MX6*, *ypt7::natNT2* |
| Ape1-GFP/ Atg19-mCherry/ypt7Δ | Ape1-GFP/ Atg19-mCherry | MATa, *his3-Δ200*, *leu2-3,112*, *ura3-52*, *lys2-801*, Ape1*-GFP::HIS3MX6*, Atg19-mCherry::KANMX, *ypt7::natNT2* |
| Ape1-GFP/ Atg19Δ /ypt7Δ | Ape1-GFP/ Atg19Δ | MATa, *his3-Δ200*, *leu2-3,112*, *ura3-52*, *lys2-801*, Ape1*-GFP::HIS3MX6*, Atg19*::natNT2*, *ypt7::natNT2* |
| Ams1-GFP/ Atg19-mCherry/ ypt7Δ | Ams1-GFP/ Atg19-mCherry | MATa, *his3-Δ200*, *leu2-3,112*, *ura3-52*, *lys2-801*, Ams1*-GFP::HIS3MX6*, Atg19-mCherry::KANMX, *ypt7::natNT2* |
| Atg19-GFP/ Ape1-mCherry/ ypt7Δ | Atg19-GFP/ Ape1-mCherry | MATa, *his3-Δ200*, *leu2-3,112*, *ura3-52*, *lys2-801*, Atg19*-GFP::HIS3MX6*, Ape1-mCherry::KANMX, *ypt7::natNT2* |
| Nuf2-GFP | MKY0217 | MATα, *his3-∆200, leu2-3,112 ura3-52, lys2-801, NUF2-EGFP::HIS3MX6* |
